# Heart rate variability reveals graded task difficulty effects and sensitization dynamics in anticipatory psychological stress via time-domain analysis

**DOI:** 10.1186/s40101-025-00413-7

**Published:** 2025-11-24

**Authors:** Ziqi Jian, Jingshi Huang, Feng Shi, Yoshihiro Shimomura

**Affiliations:** 1 Humanomics Laboratory, Graduate School of Science and Engineering, Chiba University, 1-33 Yayoi-cho, Inage Ward, Chiba City, Japan; 2 Humanomics Science Center, International Institute of Creative Design, Shanghai University of Engineering Science, 350 Xianxia Road, Changning District, Shanghai, China

**Keywords:** Anticipatory psychological stress response, Heart rate variability, SDNN, RMSSD, Mental arithmetic task

## Abstract

**Background:**

Mental arithmetic tasks effectively induce psychological stress responses, but anticipatory stress responses before task onset are often overlooked. This study investigates how task difficulty influences anticipatory stress through heart rate variability time-domain analysis.

**Methods:**

This study developed a standardized mental arithmetic task program using Unity, incorporating low, medium, and high levels by adjusting the amount of calculation and time limits. The participants were 12 healthy graduate and doctoral students. During the experiment, heart rate variability time indicators and the average RR interval were used as key physiological indicators to quantify psychological stress response. After the experiment, the participants were asked to complete the NASA Task Load Index (NASA-TLX) questionnaire to assess their workload.

**Results:**

The NASA-TLX scores revealed significant differences in perceived workload among the three levels of task difficulty. The results indicated that task difficulty had a significant impact on anticipatory psychological stress response. High-level tasks elicited significantly greater anticipatory psychological stress responses compared to low-level tasks. Among the indicators used, the standard deviation of normal-to-normal (SDNN) intervals demonstrated particularly strong performance and may serve as a reliable and sensitive measure of anticipatory psychological stress response.

**Conclusions:**

This study provides preliminary evidence supporting the use of SDNN as a complementary physiological indicator of anticipatory psychological stress responses. The findings suggest that task difficulty not only modulates individuals’ anticipatory psychological responses on a cognitive level but also significantly shapes the dynamic trajectory of the SDNN during stress development. The observed sensitization effect indicates that higher-difficulty tasks can lead to enhanced anticipatory psychological stress responses in subsequent tasks. These results have potential implications for optimizing psychological stress response intervention strategies and for the development of standardized and replicable paradigms for anticipatory psychological stress research. Future studies should incorporate a larger and more diverse sample to further investigate how individual differences influence anticipatory psychological stress responses.

## Introduction

Stress response is the body’s physiological and psychological response to perceived threats to homeostasis and is aimed at maintaining internal balance. During stress response, individuals experience changes in the activity of both the sympathetic nervous system (SNS) and the parasympathetic nervous system (PNS), along with activation of the hypothalamic‒pituitary‒adrenal (HPA) axis and the immune system [1–3]. An increase in psychological stress response levels can negatively impact cognitive performance [4, 5] and lead to an elevated heart rate, increased blood pressure, and the suppression of immune function [6]. Stressors can be categorized into physiological and psychological types, depending on the nature of the stressor [7]. Physiological stressors are typically triggered by physical stimuli, such as pain or intense exercise, whereas psychological stressors arise from adaptive autonomic responses to cognitive load, social pressure, or work-related stressors. Studies inducing psychological stress responses are valuable for identifying causal relationships between stressors and specific physiological or psychological responses, contributing significantly to our understanding of how stress response affects health, behavior, and emotions. In experimental settings, physiological stress response is often induced through physical challenges such as exercise or the Cold Pressor Test [8], whereas psychological stress response can be evoked via psychological stress paradigms such as the Trier Social Stress Test or cognitive load tasks such as mental arithmetic [9, 10].

Importantly, psychological stress responses can occur not only when a person faces a stressful situation but also in anticipation of it [11, 12]. This is because humans have the ability to anticipate future events and, on the basis of past experiences, prepare mentally, behaviorally, and physiologically [13]. This form of stress response, triggered by the anticipation of potential events, is referred to as anticipatory psychological stress response [14]. A common setting for anticipatory psychological stress response in psychology is magnetic resonance imaging (MRI), with cortisol being the most frequently used physiological marker for quantifying this type of stress response [14, 15]. Some studies have shown that stress response-inducing tasks performed in MRI environments do not result in significant changes in cortisol levels [16]. One possible explanation is that participants may have already experienced high levels of anticipatory psychological stress response before the MRI scan [17–19]. However, it should be noted that cortisol, as a physiological indicator of psychological stress response levels, may have a time lag of 5–20 min in its measurement results [20], which could lead to inaccurate measurements in rapidly changing stressors. Furthermore, the measurement of cortisol typically requires the collection, storage, and analysis of saliva, blood, or urine samples, a process that is relatively complex [21].

Compared with cortisol, HRV is an effective measure of psychological stress response. HRV offers high sensitivity in detecting stress response, allows real-time monitoring, and simplifies data collection and analysis [22, 23]. HRV is calculated on the basis of variations in the time intervals between successive heartbeats, as recorded by electrocardiogram (ECG) signals. Methods for quantitatively assessing stress response via HRV include frequency-domain analysis, time-domain analysis, and nonlinear analysis [24–26]. Frequency domain indicators of the HRV determine the dynamic weights of the SNS and PNS by analyzing the frequency of heartbeat intervals via ECG. Although this method was developed in the 1970 s and became widely applied in the 1980 s, recent studies have criticized it, suggesting that the SNS is not the sole source of low-frequency power in the ECG. In fact, PNS activity may contribute significantly to low-frequency variability, potentially biasing estimates of sympathetic activity during stress response assessment [27]. Nonlinear measures, such as approximate entropy, sample entropy, and correlation dimension, reveal the complexity and irregularity of heart rate dynamics, but their physiological significance and relationship with autonomic nervous activity remain unclear [28, 29]. In contrast, HRV time-domain indicators are simpler to calculate and easier to interpret [30], offering sensitive reflections of short-term HRV fluctuations that help assess the autonomic nervous system’s (ANS) rapid responses to various stimuli [31]. Commonly used time-domain indicators in psychological stress response evaluation include the standard deviation of NN intervals (SDNN) and the root mean square of differences between successive NN intervals (RMSSD). Additionally, the average R-R interval (AVRR) is frequently employed as a reference indicator of SNS activation in numerous studies [32–34]. While HRV time-domain indicators offer clear advantages in reflecting psychological stress response, some indicators remain controversial. For example, in a meta-analysis of 12 studies published between 2004 and 2014, Castaldo et al. noted inconsistent trends in SDNN changes under stress response [2]. Some studies have reported a decrease in SDNN with increased psychological stress response [33–35], whereas others have reported an increase [36, 37]. Schubert and Adjei suggested that this rise in SDNN may be linked to respiratory changes or the participants’ failure to reach a steady state. However, considering anticipatory psychological stress response research, these inconsistencies may be attributed to insufficient consideration of anticipatory psychological stress response in experimental designs.

In traditional psychological stress experiments, conventional approaches have focused primarily on psychological and physiological responses during or after a stressor. Experimental designs typically follow a “baseline–task–recovery” framework, in which the baseline phase is generally assumed to reflect a relaxed state of the participants. However, before the formal start of the experiment, participants are often required to complete consent forms and familiarize themselves with the procedures. For example, they may learn how to interact with experimental software before tasks such as the Montreal Imaging Stress Task or the Stroop task [38, 39] or receive instructions on the format and topic of a public speaking task [37]. These preparatory steps may induce anticipatory psychological stress responses. Additionally, the unfamiliar laboratory environment, along with the physical presence of electrodes and wires attached to the participants, may further exacerbate anticipatory psychological stress responses [40]. Owing to inherent limitations in experimental design, it is difficult to completely eliminate anticipatory psychological stress response effects, especially when participants are required to perform under multiple conditions. In such cases, it is challenging to avoid anticipatory psychological stress responses influencing later conditions after the initial exposure. This anticipatory psychological stress response may hinder participants from reaching a truly relaxed state during the baseline phase, thereby exerting a profound influence on subsequent data patterns. In previous studies, researchers have often used randomization of task order to control for the potential impact of anticipatory psychological stress responses and to mitigate data biases [41]. Although this approach offers certain methodological advantages, it overlooks potential individual differences between participants’ subjective psychological preparation for the upcoming task and their physiological responses. In other words, randomization of task order does not eliminate the influence of anticipatory psychological stress responses; rather, it averages out its effects, which may obscure critical individual differences and mechanistic insights. Therefore, instead of relying solely on passive control strategies, actively and accurately measuring anticipatory psychological stress responses may represent a more forward-looking and meaningful solution to this issue. By quantifying individuals’ psychological and physiological responses prior to task onset, researchers can gain deeper insights into the temporal dynamics and individualized characteristics of stress responses, thereby enhancing the scientific rigor and interpretability of psychological stress research.

To address key challenges in current anticipatory psychological stress research effectively, the present study developed a structured and highly controllable experimental paradigm. We designed three mental arithmetic tasks with varying levels of difficulty, including a widely used classic continuous decrement task and two independently developed arithmetic programs with clearly defined and controllable difficulty levels. By systematically introducing task difficulty as a core variable and collecting HRV time-domain indicators—AVRR, SDNN, and RMSSD—both prior to task onset and during task execution, this study aimed to comprehensively examine the effects of task difficulty on psychological and anticipatory psychological stress response. This study not only focused on the dynamic changes in stress response across different temporal phases but also evaluated the responsiveness and applicability of multiple HRV indicators at each phase. Through this approach, we sought to elucidate the underlying mechanisms of anticipatory psychological stress response and deepen our understanding of individuals’ psychological and physiological states prior to task engagement. The experimental design proposed in this study offers a solid theoretical and practical foundation for the future development of standardized and replicable paradigms for anticipatory psychological stress research while also providing valuable insights for optimizing psychological stress response intervention strategies.

## Methods

### Participants

The participants in this study were 15 current graduate students, aged between 24 and 34 years, all with no history of cardiovascular disease. Prior to inclusion in the experiment, each participant underwent a math ability screening test to ensure that individuals with excessively high or low calculation skills were excluded. To ensure the accuracy of the experimental results, all participants were required to provide adequate sleep the night before the experiment and to refrain from smoking or consuming any stimulants, including coffee and tea, for at least 12 h. To further ensure data reliability, all experiments were conducted between 1:00 p.m. and 3:00 p.m. Additionally, none of the participants reported any history of heart disease or other chronic conditions. Due to excessive physiological signal artifacts primarily caused by electromagnetic interference and motion-related ECG noise, data from three participants were excluded after preprocessing and visual inspection. These signals exhibited irregular RR intervals and waveform distortions that could not be sufficiently corrected through filtering or artifact removal, resulting in a final sample size of 12 participants (4 females and 8 males; average age = 29 years).

Before the experiment began, the participants were provided with detailed written informed consent forms and were ensured a full understanding of the experimental process, objectives, and potential risks. Each participant received a compensation of 4000 yen for their involvement. This study was reviewed and approved by the Ethics Committee of Chiba University (Ethics Review No. R4-22), ensuring compliance with ethical standards and regulations.

### Experiments

**Fig. 1:**
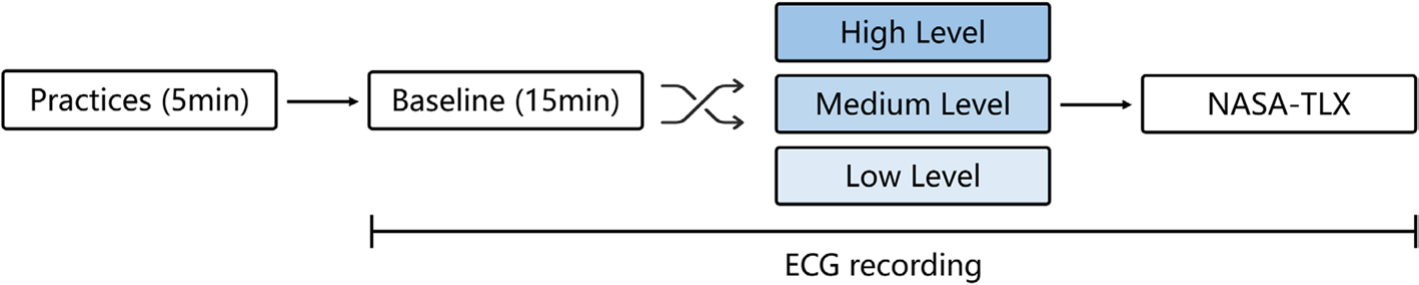
Experimental procedure

The experiment was conducted in a temperature-controlled room set to approximately 25 °C, which was soundproof and electromagnetically shielded to ensure the environmental stability and accuracy of the data. Upon arriving at the lab, participants were given a thorough briefing on the experimental environment, the equipment used, task requirements, study objectives, and potential risks and benefits. They then practiced using the mental arithmetic software to ensure that they could perform the tasks efficiently during the experiment. The participants sat in front of a computer screen in the position where they were most comfortable and remained seated throughout the experiment. As shown in Fig. 1, the experimental procedure began with a 15-min rest phase, during which 5 min of data were collected as baseline measurements. The participants subsequently completed a mental arithmetic task whose actual duration varied between 15 and 20 min, depending on individual response speed and error rates. To ensure consistency across participants, only the first 15 min of data were used for HRV analysis. After the task, they had another 15-min rest phase. To minimize the impact of altered breathing patterns, which can affect vagal activity and distort HRV measures, participants were instructed to interact with the task using a mouse and keyboard. Unlike oral responses, which require speaking and can disrupt respiratory rhythm [10, 42], this input method avoids speech-related respiratory artifacts and ensures more stable physiological recordings. After the experiment, the participants were asked to complete the NASA Task Load Index (NASA-TLX) questionnaire to assess their workload [43].

**Fig. 2:**
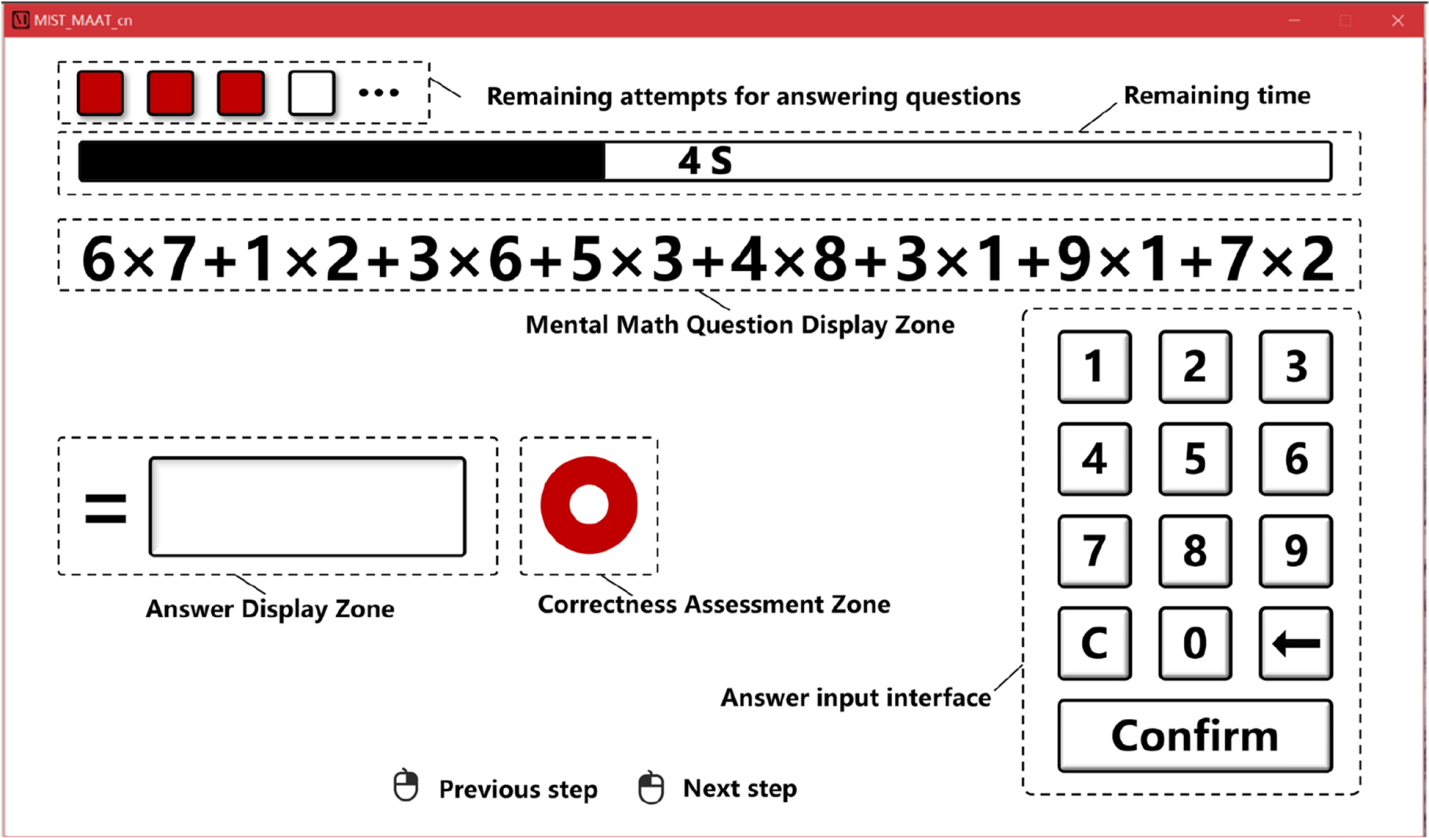
Program interface for mental arithmetic

To achieve precise control over the difficulty levels of the mental arithmetic tasks, this study followed the design principles of the Montreal Imaging Stress Task and developed a standardized, computerized arithmetic task program using Unity. As shown in Fig. 2, the program interface consists of several key functional areas: the mental math question display zone presents the current mental arithmetic problem, and the answer input interface allows participants to enter their responses. The correctness assessment zone provides immediate feedback on whether the answer is correct, while a countdown bar indicates the remaining time, and the remaining attempts to answer the questions show how many problems the participants can still answer during the current task. Each arithmetic task consists of several calculation units, with each unit involving the multiplication of two single-digit numbers. The difficulty level of the task is determined by the number of calculation units involved. To minimize the potential negative impact of incorrect answers on participants, the program is set to a unified feedback mode, where the feedback is always presented correctly, regardless of whether the participant’s answer is accurate. The program backend limits the time allowed for each calculation, requiring participants to complete the calculation within the specified time frame. If a participant fails to respond within the time limit, the question is marked as incorrect. Additionally, there is a maximum number of timeouts allowed per task; once a participant reaches this threshold, the experiment automatically terminates, indicating that the participant is unable to continue with subsequent tasks.

In this experiment, participants perform mental arithmetic tasks with three different levels of difficulty. The specific experimental conditions were as follows:

- Low level: Conducting a Word document, participants are required to perform continuous decremental calculations by subtracting 7 for 15 min (e.g., 10,000−7 = 9993, 9993−7 = 9986). Before the task begins, participants are informed that they do not need to focus on whether their answers are correct.
- Medium level: Conducted within the arithmetic program, each problem consists of the sum of three calculation units, with a 30-s time limit per question. The maximum number of timeouts is set at 8, and each participant is required to perform calculations for 15 min. If a participant exceeds eight timeouts, it is defined as a failure to complete the experiment.
- High level: Also conducted within the arithmetic program, each problem consists of the sum of six calculation units, with a 45-s time limit per question. The maximum number of timeouts is set at 8, and participants must perform calculations for 15 min. Exceeding eight timeouts is defined as a failure to complete the experiment.

For the low-level task, which is designed as a continuous decrement of 7 in a Word document, participants might have noticed calculation errors during the task, which could cause them to feel tense. To prevent any additional stressor, the participants were explicitly informed before the experiment that they did not need to focus on the correctness of their calculations. Consequently, the error rate data for the low-level task was not included in the statistical analysis.

To prevent fatigue from consecutive experiments, each participant participated in only one difficulty level task per day. All the participants completed experiments for the three different difficulty level tasks, with at least 24 h between each task. To control for potential order effects, the sequence of high- and low-level tasks was randomly balanced across participants, whereas the medium-level task was always performed in the middle.

### Physiological signal interpretation and statistical analysis

This study used HRV time-domain indicators as markers of stress response. ECG data were collected via the MP160 system’s wireless BioNomadix ECG module, ensuring both convenience and accuracy in data acquisition. The ECG electrodes were placed according to the standard three-lead placement method to obtain high-quality ECG signals. The collected ECG data were preprocessed via AcqKnowledge4.2 software, with a sampling rate of 1000 Hz. To eliminate motion artifacts and abnormal RR intervals, the ECG data were subjected to 5-point smoothing filtering, and further data purification was performed via a threshold method, excluding RR intervals shorter than 0.5 s and longer than 1.5 s. After preprocessing, Kubios HRV Premium software was used to calculate the key HRV time-domain indicators, including the AVRR, SDNN, and RMSSD. These indicators provide essential physiological insights for assessing psychological stress response [44].

The AVRR data, measured in milliseconds, was calculated via the following formula:

$$\text{AVRR}=\frac{1}{n}\sum\limits _{i=1}^{n}{\text{RR}}_{i}$$

where *RR*_i_ represents the *i*-th RR interval and *n* represents the total number of RR intervals.

The SDNN data, measured in milliseconds, was calculated via the following formula:

$$\text{SDNN}=\sqrt{\frac1{n-1}\sum\limits_{i=1}^n\left({\mathrm{RR}}_{\mathrm i}-\overline{\mathrm{RR}}\right)^2}$$

The RMSSD data, measured in milliseconds, was calculated via the following formula:

$$\text{RMSSD}=\sqrt{\frac1{n-1}\sum\limits_{i=1}^{n-1}\left({\mathrm{RR}}_{\mathrm i+1}-{\mathrm{RR}}_{\mathrm i}\right)^2}$$

The mental arithmetic task program recorded the error rates of all participants in the medium- and high-level tasks, and statistical analysis was conducted via nonparametric tests for paired samples. Additionally, to assess task load, the NASA-TLX was employed, and the results of the scale were analyzed via one-way analysis of variance (ANOVA).

To investigate the differences in the effects of various difficulty levels of mental arithmetic tasks on AVRR, SDNN, and RMSSD data during the baseline and experimental phases, the study utilized IBM SPSS Statistics 25 for a repeated measures two-way ANOVA with a 3 (task difficulty: low, medium, high) × 2 (time phase: baseline, experimental) design. This approach was employed to compare the potential impacts of different task levels on the HRV data. Prior to analysis, all the HRV data were subjected to the Shapiro–Wilk test to assess normality. Additionally, Mauchly’s test of sphericity was conducted to ensure that the assumptions for the analysis were met. For data that met the sphericity assumption, Greenhouse–Geisser corrections were applied; for those that did not, the Birkhoff trajectory correction was used to ensure the accuracy of the multivariate. The statistical significance level was set at 0.05 in all analyses. The symbol (*) indicates significant differences between conditions (*p* < 0.05). The symbol (+) indicates tendency differences between conditions (*p* < 0.1).

## Results

### NASA-TLX scale and behavioral results

**Fig. 3:**
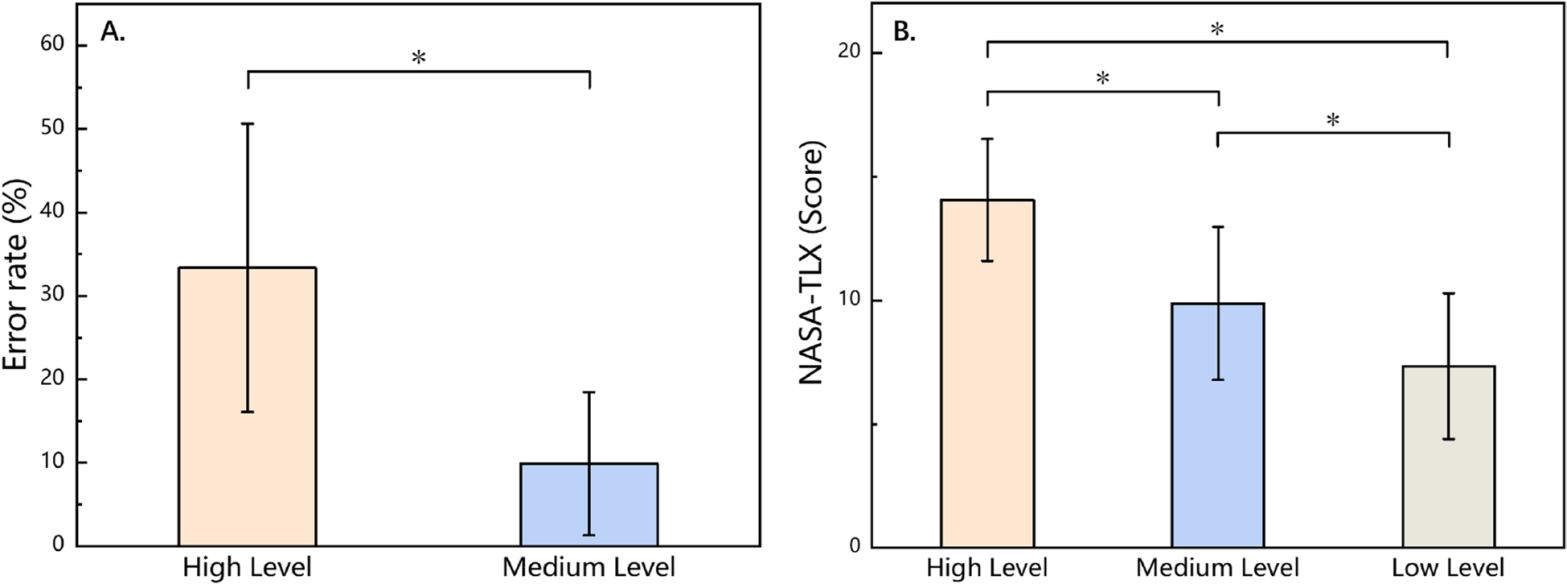
Behavioral outcomes under mental arithmetic tasks of varying levels of difficulty. **A** Error rate of mental arithmetic. **B** NASA-TLX workload

This study compared the error rates of participants in high-level and medium-level mental arithmetic tasks. Descriptive statistics revealed that the average error rate in the high-level group was 33.06%, with a standard deviation of 15.59%, whereas the medium-level group had an average error rate of 9.88%, with a standard deviation of 8.42%. Given that the error rate data did not meet the assumption of a normal distribution, a nonparametric Wilcoxon rank-sum test was used to analyze the differences between the two groups. The results, as shown in Fig. 3A, indicate that the error rate in the high-level task was significantly higher than that in the medium-level task (*p* < 0.05).

The NASA-TLX scores for the three different difficulty levels of mental arithmetic tasks were confirmed to follow a normal distribution on the basis of the Shapiro–Wilk normality test. As shown in Fig. 3B, the mean and standard deviation of the workload scores for each difficulty level task were as follows: the high-level group had a mean score of 13.95 with a standard deviation of 3.41, the medium-level group had a mean score of 10.42 with a standard deviation of 3.36, and the low-level group had a mean score of 7.05 with a standard deviation of 1.61. One-way ANOVA revealed significant differences in the mean scores across the groups (*F* (2, 33) = 13.943, *p* < 0.05). To further identify where the differences lay, post hoc tests were conducted. The results revealed significant differences between the high- and medium-level groups (*p* < 0.05), between the high- and low-level groups (*p* < 0.05), and between the medium- and low-level groups (*p* < 0.05). These findings suggest that the task difficulty level had a significant effect on the participants’ perceived workload.

### SDNN

The results of the two-way repeated-measures ANOVA revealed that the SDNN data during the baseline phase for the high-, medium-, and low-level tasks were 35.60 ± 9.68 ms, 41.71 ± 8.07 ms, and 54.47 ± 9.43 ms, respectively. During the tasks phase, the SDNN values for the high-, medium-, and low-level tasks were 48.89 ± 14.07 ms, 48.12 ± 11.20 ms, and 46.17 ± 12.16 ms, respectively.

The main effect of the experimental phase was not significant. However, the main effect of task difficulty level was significant (*F* (2, 44) = 7.82, *p* < 0.05, *η*^2^ = 0.262). Additionally, there was a significant interaction effect between the experimental phase and task difficulty level (*F* (2, 44) = 14.223, *p* < 0.05, *η*^2^ = 0.393), indicating that the trend of SDNN changes between the baseline and tasks phase differed significantly across tasks with different difficulty levels. As a result, a separate effects analysis was conducted to examine these differences further.

**Fig. 4:**
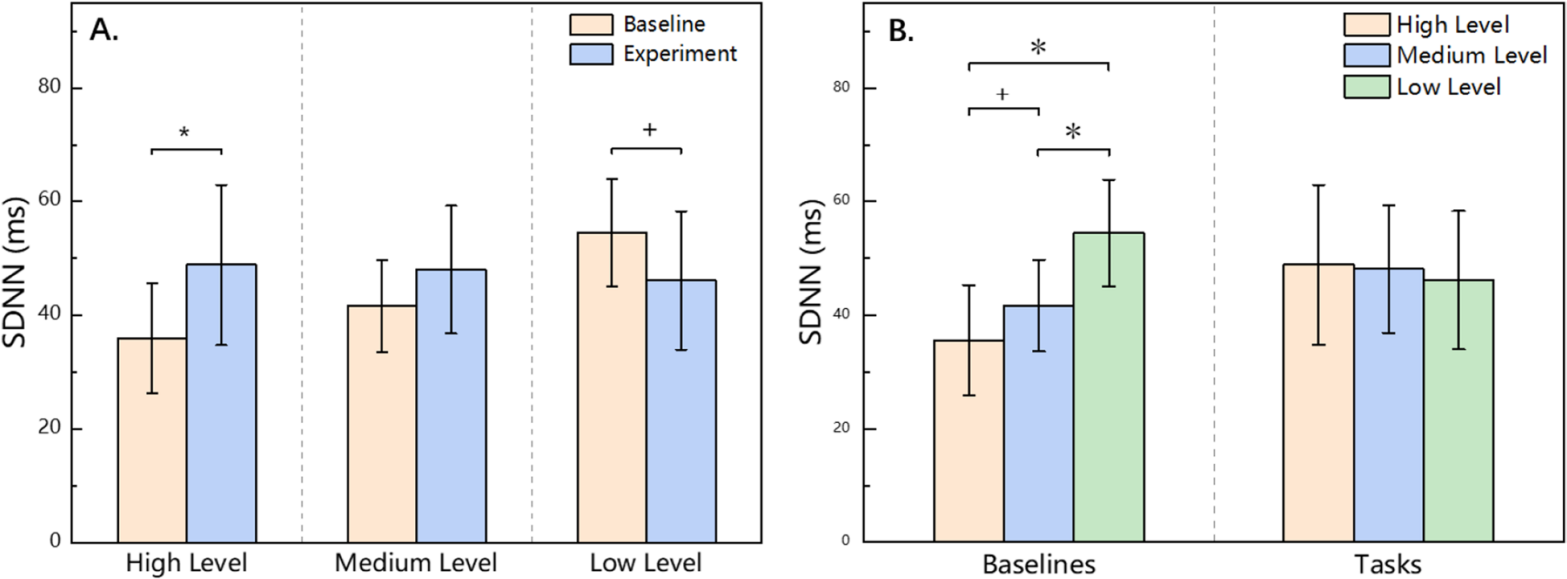
Results of multiple comparisons of SDNN data for task difficulty × experimental phase

The simple effect results for within-group comparisons during the experimental phase are shown in Fig. 4A. In the high-level group, the baseline phase values were lower than those during the task phase, although the difference only tended to show differences (*p* < 0.1). In the medium-level group, there was no significant difference between the baseline and task phase values. In the low-level group, the baseline phase values were significantly greater than those during the task phase (*p* < 0.05).

The simple effect results for between-group comparisons of task difficulty level are shown in Fig. 4B. In the baselines phase, the high-level group had lower SDNN values than did the medium-level group, with the result tendency differences (*p* < 0.1). The high-level group had significantly lower SDNN values than did the low-level group (*p* < 0.05). The medium-level group also had significantly lower SDNN values than did the low-level group (*p* < 0.05). During the tasks phase, there were no significant differences in the SDNN values among the high-, medium-, and low-level tasks.

### AVRR

The two-way repeated measures ANOVA results indicate that the average AVRR data during the baselines phase was 799.43 ± 135.66 ms for the high-level task, 767.07 ± 120.47 ms for the medium-level task, and 814.85 ± 121.23 ms for the low-level task. During the tasks phase, the AVRR data were 765.64 ± 112.11 ms for the high-level task, 744.71 ± 103.11 ms for the medium-level task, and 797.44 ± 417.43 ms for the low-level task.

**Fig. 5:**
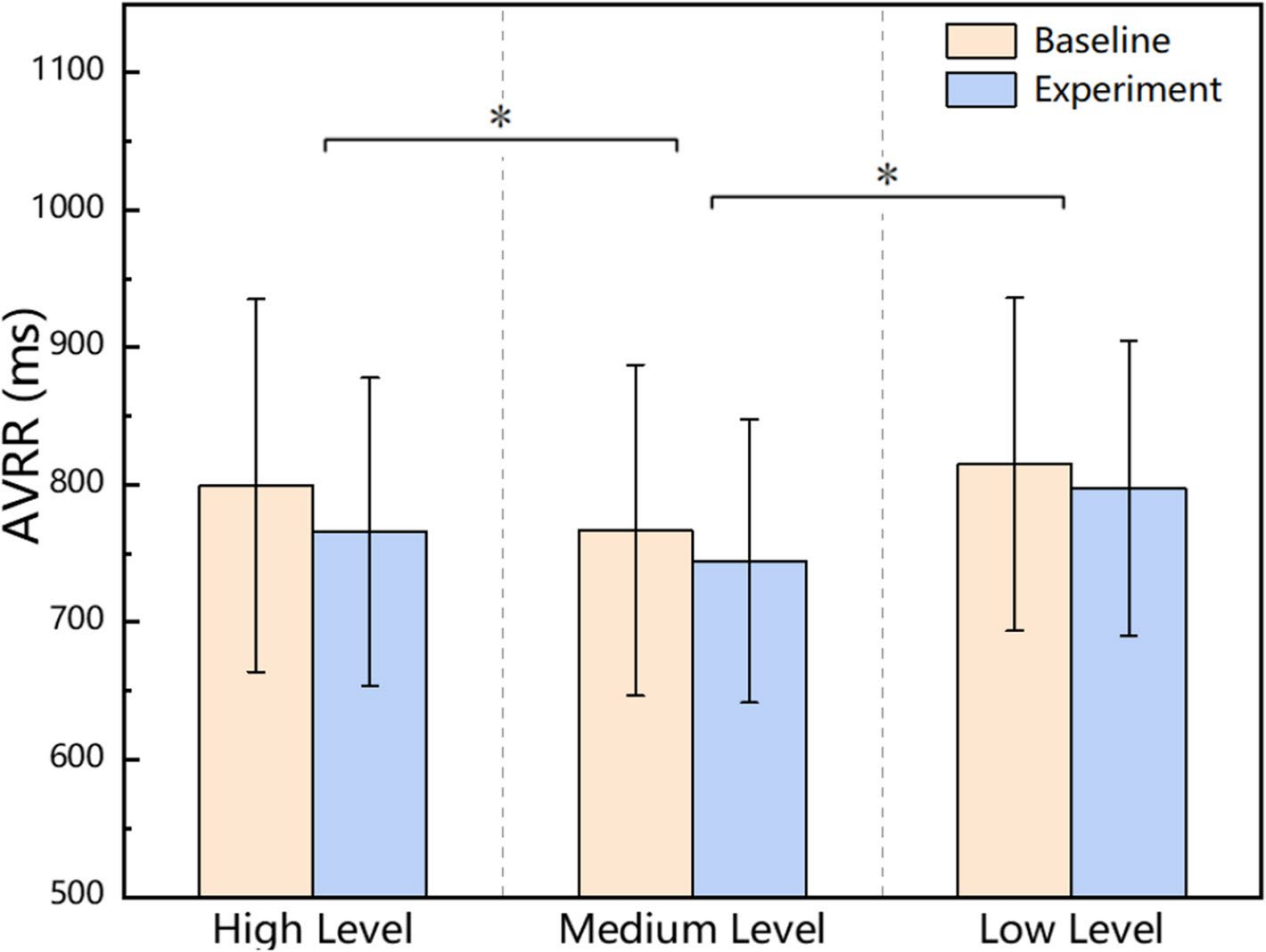
Results of multiple comparisons of AVRR data for task difficulty × experimental phase

The main effect of the experimental phase was not significant (*F* (1, 22) = 0.282, *p* > 0.05, *η*^2^ = 0.013), whereas the main effect of task difficulty was significant (*F* (2, 44) = 11.084, *p* < 0.05, *η*^2^ = 0.335). The interaction effect between experimental phase and task difficulty was not significant (*F* (2, 44) = 0.309, *p* > 0.05, *η*^2^ = 0.014). Accordingly, post hoc comparisons were performed on the phase-collapsed AVRR values. These analyses revealed that the medium-level task elicited significantly lower AVRR than both the high-level (*p* < 0.05) and low-level tasks (*p* < 0.05), while no significant difference was found between the high- and low-level tasks (*p* > 0.05) (as shown in Fig. 5).

### RMSSD

The two-way repeated measures ANOVA results indicate that the average RMSSD data during the baselines phase was 29.00 ± 12.68 ms for the high-level task, 28.92 ± 10.76 ms for the medium-level task, and 31.76 ± 9.38 ms for the low-level task. During the tasks phase, the AVRR data was 30.96 ± 13.01 ms for the high-level task, 30.96 ± 9.94 ms for the medium-level task, and 33.83 ± 11.05 ms for the low-level task.

The two-way repeated-measures ANOVA showed that the main effect of experimental phase (baseline vs. task) was not significant (*F* (1, 22) = 0.253, *p* > 0.05, *η*^2^ = 0.011). The main effect of task difficulty was also not significant (*F* (2, 44) = 1.507, *p* > 0.05, *η*^2^ = 0.064). Furthermore, the interaction effect between phase and task difficulty was not significant (*F* (2, 44) = 0.000, *p* > 0.05, *η*^2^ = 0.000). Accordingly, no post hoc comparisons were performed.

## Discussion

### Behavioral

The difficulty grading of the mental arithmetic tasks in this study was successful, as demonstrated by the NASA-TLX workload ratings shown in Fig. 3B, which indicate significant differences in workload among the three levels of task difficulty [43]. Additionally, as shown in Fig. 3A, participants’ accuracy in the high-level task was significantly lower than that in the medium-level task, indicating that increased difficulty directly affects performance. Importantly, although the error rates were significantly different, the system provided correct feedback even if the participants submitted incorrect answers during the experiment. This design ensured that participants were not negatively impacted by incorrect feedback, allowing for a focus on the effects of task difficulty level on their stress levels while avoiding additional psychological burden from erroneous feedback.

### SDNN

In this experiment, mental arithmetic tasks with varying difficulty levels successfully elicited significant behavioral differences and revealed complex changes in HRV indicators. As shown in Fig. 4A, from the baseline phase to the task phase, the SDNN exhibited distinct trends across the three difficulty levels: it increased significantly in the high-level task, showed no significant change in the medium-level task, and decreased in the low-level task. According to established theoretical frameworks, an increase in psychological stress response typically activates the SNS, promoting the release of norepinephrine. Norepinephrine binds to *β*-adrenergic receptors in the heart, accelerating myocardial cell depolarization and thereby increasing heart rate [45]. Given the close relationship between SDNN and heart rate, an elevated heart rate is often accompanied by a decrease in HRV, resulting in a lower SDNN. Conversely, when heart rate decreases, SDNN generally increases. This relationship—where a rise in heart rate induces a reduction in HRV—is referred to as cycle length dependence [46]. However, the present findings did not fully align with this expected pattern. In particular, under the high-level condition, SDNN did not decrease; rather, it increased significantly from the baseline to the task phase. On the surface, this phenomenon appears to contradict existing theory, but its underlying mechanism may be linked to the role of anticipatory psychological stress response. During the baseline phase of the high-level task, participants may have anticipated that their computational abilities would be insufficient to meet the upcoming challenge, thereby generating a pronounced anticipatory psychological stress response. This response could have enhanced SNS activity in advance, leading to an abnormally low baseline SDNN. Once the task commenced, gradual adaptation to the task may have alleviated psychological stress, resulting in a rebound in SDNN values. In contrast, during the baseline phase of the low-level task, anticipatory psychological stress response was relatively low, and SDNN remained at normal or elevated levels. As the task began, a slight increase in psychological stress response likely led to the typical downward trend in SDNN. For the medium-level task, the anticipatory psychological stress response during the baseline phase was relatively similar in magnitude to the psychological stress response induced while performing the task, resulting in no significant change in SDNN.

To further verify the effect of task difficulty on anticipatory psychological stress response, this study compared participants’ baseline SDNN values across different task difficulty groups. As shown in Fig. 4B, the baseline SDNN in the high-level task group was significantly lower than that in the medium-level group, whereas the baseline SDNN in the low-level group was significantly higher than that in the medium-level group. This pattern further supports the hypothesis that “the higher the task difficulty, the stronger the anticipatory psychological stress response it elicits” and is consistent with previous findings. For example, van Paridon et al. reported that cortisol levels in skydivers prior to competition were significantly higher than those in athletes participating in other sports, indicating that highly challenging tasks are more likely to trigger intense anticipatory psychological stress responses [47]. Although that study did not use HRV indicators, it nevertheless provided empirical support for the association between task difficulty and anticipatory psychological stress response through the use of cortisol as a psychological stress response biomarker. In addition, De Rivecourt et al. found in a flight simulation experiment that as task difficulty increased, participants reported significantly higher subjective stress response levels, accompanied by accelerated heart rate and decreased SDNN [48]. These results further indirectly confirm the modulatory role of task difficulty in psychological stress responses and their physiological correlates, thereby providing both theoretical and physiological support for the present findings.

It is noteworthy that although significant differences in SDNN values were observed between groups during the baseline phase, these differences were no longer significant during the task phase. This phenomenon suggests that once the task began, participants’ psychological stress responses appeared to converge across conditions, thereby diminishing the between-group differences originally induced by task difficulty. Two possible explanations are proposed. First, for the high- and medium-level tasks, participants had already experienced considerable neural activation during the baseline phase. This may have limited the extent to which the nervous system could be further activated during the mental arithmetic task and, for some participants, may have led to a gradual adaptation to the stressor, resulting in a reduction in stress responses. Several studies have confirmed this phenomenon. For example, in a meta-analysis of competitive athletes, van Paridon et al. reported that cortisol levels typically rose significantly before competition but did not further increase during actual performance [47]. Similarly, Storniolo et al. found in a sprinting task that after completing a 60-m maximal sprint, athletes’ HRV entered a “plateau phase” in which ANS activity remained stable for a short period after peaking, potentially reflecting a characteristic of acute stress responses [49]. Second, for the low-level task, the anticipatory psychological stress response during the baseline phase was relatively low. However, the increase in cognitive load during the mental arithmetic task may have elevated psychological stress levels during the task phase. This increase may have caused the SDNN values in the low-level group to approach those of the medium- and high-level groups, thereby reducing between-group differences.

In conclusion, task difficulty not only modulates individuals’ anticipatory psychological responses on a cognitive level but also significantly shapes the dynamic trajectory of the SDNN during stress development. By introducing the SDNN as a sensitive physiological indicator, this study reveals a systematic relationship between task difficulty and anticipatory psychological stress responses, providing new empirical support and a theoretical foundation for understanding the mechanisms underlying stress responses.

### AVRR

As shown in Fig. 5, the main effect of task difficulty was significant, with post hoc comparisons indicating that the medium-level task elicited significantly lower AVRR than both the high- and low-level tasks, whereas no significant difference was found between the high- and low-level tasks. This result suggests that AVRR primarily reflects overall heart rate levels and can differentiate between different task workload conditions. Notably, the lowest AVRR observed in the medium-level task may be related to a sensitization effect. The sensitization effect refers to the phenomenon whereby, after exposure to a particularly strong stressor, individuals exhibit an enhanced stress response to subsequent similar stressors [50]. Belda et al. further noted that the stressor must be sufficiently intense or threatening, and that the degree of sensitization is closely related to the duration of exposure [51]. In the present experiment, the medium-level task was always performed on the second day. For participants who had already performed the high-level task on the first day, the strong initial stressor may have markedly amplified their anticipatory stress response to the medium-level task on the second day through sensitization, thereby leading to decreased AVRR in the medium-level condition.

The nonsignificant main effect of experimental phase and interaction effect suggests that although AVRR has some utility in reflecting sympathetic activation and distinguishing overall task workload levels, its discriminative power is limited under short-term stress and anticipatory stress conditions. Considering the combined influence of sensitization and anticipatory stress, AVRR may be more appropriate as a reference indicator of overall workload levels rather than as a marker of dynamic stress changes across experimental phases.

### RMSSD

Compared with SDNN, RMSSD did not exhibit the same level of sensitivity in detecting anticipatory psychological stress responses. Neither the main effect of task difficulty nor the main effect of experimental phase was significant. This finding contrasts with previous studies reporting a general consistency between RMSSD and SDNN in assessing stress responses [52, 53]. A plausible explanation is that RMSSD primarily reflects short-term beat-to-beat variability dominated by parasympathetic activity [54, 55]. Under anticipatory stress, simultaneous activation of the SNS and PNS may occur [56, 57], and such co-activation could mask changes in RMSSD, thereby reducing its sensitivity relative to SDNN.

### Limitation

This study has several limitations. First, the participant sample was relatively small and homogeneous, and did not include key demographic variables such as age and cultural background, which limit the generalizability of the findings. Second, the absence of a fully relaxed baseline phase may have affected the precision of the comparisons between the baseline and experimental phases. Future research should include more representative samples, incorporate individuals who are unaffected by any external factors during the resting phase, and further explore how individual differences influence anticipatory psychological stress responses. A series of follow-up experiments is currently being planned to address these limitations and expand upon the present findings.

## Conclusion

This study examined the effects of task difficulty, HRV time-domain indicators, and task order on anticipatory psychological stress responses via a mental arithmetic paradigm with controlled difficulty levels. The results revealed the following:

1. Increased arithmetic difficulty significantly elevated participants’ anticipatory psychological stress responses before task initiation.
2. Among the three HRV time-domain indicators, the SDNN exhibited better sensitivity and stability than the AVRR and RMSSD did, indicating its greater suitability as a physiological marker of anticipatory psychological stress responses. This phenomenon has not been sufficiently confirmed in previous studies.
3. Higher-difficulty arithmetic tasks triggered a sensitization effect, such that participants exhibited stronger anticipatory psychological stress responses in subsequent arithmetic tasks.

Although cortisol has long been widely used as a core biomarker for assessing anticipatory psychological stress responses, the potential value of the SDNN as an accessible and noninvasive HRV indicator has received relatively limited attention in this context. This study provides preliminary evidence supporting the use of SDNN as a complementary physiological indicator of anticipatory psychological stress responses. In future studies, it could be used in combination with traditional biomarkers such as cortisol to improve the sensitivity and practical applicability of anticipatory psychological stress responses assessment.

## Data Availability

The datasets used and analysed during the current study are available from the corresponding author on reasonable request.

## Abbreviations

HRV: Heart rate variability
ECG: Electrocardiogram
AVRR: Average R-R interval
SDNN: Standard deviation of normal-to-normal intervals
RMSSD: Root mean square of successive differences
NASA-TLX: NASA Task Load Index
HPA: Hypothalamic‒pituitary‒adrenal
SNS: Sympathetic nervous system
PNS: Parasympathetic nervous system
ANS: Autonomic nervous system

## References

[1] Beauchaine TP, Thayer JF. Heart rate variability as a transdiagnostic biomarker of psychopathology. Int J Psychophysiol. 2015;98(2, Pt 2):338–50. 10.1016/j.ijpsycho.2015.08.004. 26272488 10.1016/j.ijpsycho.2015.08.004

[2] Castaldo R, Melillo P, Bracale U, Caserta M, Triassi M, Pecchia L. Acute mental stress assessment via short term HRV analysis in healthy adults: a systematic review with meta-analysis. Biomed Signal Process Control. 2015;18:370–7. 10.1016/j.bspc.2015.02.012.

[3] Hamidovic A, Van Hedger K, Choi SH, Flowers S, Wardle M, Childs E. Quantitative meta-analysis of heart rate variability finds reduced parasympathetic cardiac tone in women compared to men during laboratory-based social stress. Neurosci Biobehav Rev. 2020;114:194–200. 10.1016/j.neubiorev.2020.04.005. 32320815 10.1016/j.neubiorev.2020.04.005 PMC7422617

[4] LeBlanc VR. The effects of acute stress on performance: implications for health professions education. Acad Med. 2009;84(10 Suppl):S25–33. 10.1097/ACM.0b013e3181b37b8f. 19907380 10.1097/ACM.0b013e3181b37b8f

[5] Mandrick K, Peysakhovich V, Rémy F, Lepron E, Causse M. Neural and psychophysiological correlates of human performance under stress and high mental workload. Biol Psychol. 2016;121:62–73. 10.1016/j.biopsycho.2016.10.002. 27725244 10.1016/j.biopsycho.2016.10.002

[6] Segerstrom SC, Miller GE. Psychological stress and the human immune system: a meta-analytic study of 30 years of inquiry. Psychol Bull. 2004;130(4):601–30. 10.1037/0033-2909.130.4.601. 15250815 10.1037/0033-2909.130.4.601 PMC1361287

[7] Dickerson SS, Kemeny ME. Acute stressors and cortisol responses: a theoretical integration and synthesis of laboratory research. Psychol Bull. 2004;130(3):355–91. 10.1037/0033-2909.130.3.355. 15122924 10.1037/0033-2909.130.3.355

[8] Rubio G, et al. Stress induced by the socially evaluated cold-pressor test causes equivalent deficiencies of sensory gating in male subjects with schizophrenia and healthy controls. Psychiatry Res. 2015;228(3):283–8. 10.1016/j.psychres.2015.05.097. 26154819 10.1016/j.psychres.2015.05.097

[9] Labuschagne I, Grace C, Rendell P, Terrett G, Heinrichs M. An introductory guide to conducting the Trier Social Stress Test. Neurosci Biobehav Rev. 2019;107:686–95. 10.1016/j.neubiorev.2019.09.032. 31560923 10.1016/j.neubiorev.2019.09.032

[10] Liang CS, Lee JF, Chen CC, Chang YC. Reactive heart rate variability in male patients with first-episode major depressive disorder. Prog Neuro-Psychopharmacol Biol Psychiatry. 2015;56:52–7. 10.1016/j.pnpbp.2014.08.004. 10.1016/j.pnpbp.2014.08.004 25149628

[11] Engert V, Efanov SI, Duchesne A, Vogel S, Corbo V, Pruessner JC. Differentiating anticipatory from reactive cortisol responses to psychosocial stress. Psychoneuroendocrinology. 2013;38(8):1328–37. 10.1016/j.psyneuen.2012.11.018. 23246327 10.1016/j.psyneuen.2012.11.018

[12] Zandara M, Garcia-Lluch M, Villada C, Hidalgo V, Salvador A. Searching for a job: cardiac responses to acute stress and the mediating role of threat appraisal in young people. Stress Health. 2018;34(1):15–23. 10.1002/smi.2757. 28417549 10.1002/smi.2757

[13] Farooqui AA, Manly T. Anticipatory control through associative learning of subliminal relations: invisible may be better than visible. Psychol Sci. 2015;26(3):325–34. 10.1177/0956797614564191. 25694442 10.1177/0956797614564191

[14] Pulopulos MM, Vanderhasselt MA, De Raedt R. Association between changes in heart rate variability during the anticipation of a stressful situation and the stress-induced cortisol response. Psychoneuroendocrinology. 2018;94:63–71. 10.1016/j.psyneuen.2018.05.004. 29758470 10.1016/j.psyneuen.2018.05.004 PMC5967249

[15] Turan B. Predictors of anticipatory cortisol reactivity to subsequent stressors. Physiol Behav. 2015;149:239–46. 10.1016/j.physbeh.2015.06.011. 26071396 10.1016/j.physbeh.2015.06.011

[16] Allendorfer JB, Szaflarski JP. Contributions of fMRI towards our understanding of the response to psychosocial stress in epilepsy and psychogenic nonepileptic seizures. Epilepsy Behav. 2014;35:19–25. 10.1016/j.yebeh.2014.03.023. 24785430 10.1016/j.yebeh.2014.03.023

[17] Muehlhan M, Lueken U, Wittchen HU, Kirschbaum C. The scanner as a stressor: evidence from subjective and neuroendocrine stress parameters in the time course of a functional magnetic resonance imaging session. Int J Psychophysiol. 2011;79(2):118–26. 10.1016/j.ijpsycho.2010.09.009. 20875462 10.1016/j.ijpsycho.2010.09.009

[18] Tessner KD, Walker EF, Hochman K, Hamann S. Cortisol responses of healthy volunteers undergoing magnetic resonance imaging. Hum Brain Mapp. 2006;27(11):889–95. 10.1002/hbm.20229. 16544325 10.1002/hbm.20229 PMC6871496

[19] Thorpe S, Salkovskis PM, Dittner A. Claustrophobia in MRI: the role of cognitions. Magn Reson Imaging. 2008;26(8):1081–8. 10.1016/j.mri.2008.01.022. 18524527 10.1016/j.mri.2008.01.022

[20] Kudielka BM, Kirschbaum C. Sex differences in HPA axis responses to stress: a review. Biol Psychol. 2005;69(1):113–32. 10.1016/j.biopsycho.2004.11.009. 15740829 10.1016/j.biopsycho.2004.11.009

[21] Stalder T, Kirschbaum C, Kudielka BM, Adam EK, Pruessner JC, Wüst S, et al. Assessment of the cortisol awakening response: expert consensus guidelines. Psychoneuroendocrinology. 2016;63:414–32. 10.1016/j.psyneuen.2015.10.010. 26563991 10.1016/j.psyneuen.2015.10.010

[22] Rajendra Acharya U, Joseph KP, Kannathal N, Lim CM, Suri JS. Heart rate variability: a review. Med Biol Eng Comput. 2006;44(12):1031–51. 10.1007/s11517-006-0119-0. 17111118 10.1007/s11517-006-0119-0

[23] Shaffer F, Ginsberg JP. An overview of heart rate variability metrics and norms. Front Public Health. 2017;5:258. 10.3389/fpubh.2017.00258. 29034226 10.3389/fpubh.2017.00258 PMC5624990

[24] Castrillón CIM, Miranda RAT, Cabral-Santos C, Vanzella LM, Rodrigues B, Vanderlei LCM, et al. High-intensity intermittent exercise and autonomic modulation: effects of different volume sessions. Int J Sports Med. 2017;38(6):468–72. 10.1055/s-0042-121898. 28388782 10.1055/s-0042-121898

[25] Montano N, Ruscone TG, Porta A, Lombardi F, Pagani M, Malliani A. Power spectrum analysis of heart rate variability to assess the changes in sympathovagal balance during graded orthostatic tilt. Circulation. 1994;90(4):1826–31. 10.1161/01.cir.90.4.1826. 7923668 10.1161/01.cir.90.4.1826

[26] Vuksanović V, Gal V. Heart rate variability in mental stress aloud. Med Eng Phys. 2007;29(3):344–9. 10.1016/j.medengphy.2006.05.011. 16807051 10.1016/j.medengphy.2006.05.011

[27] Billman GE. The LF/HF ratio does not accurately measure cardiac sympatho-vagal balance. Front Physiol. 2013;4:26. 10.3389/fphys.2013.00026. 23431279 10.3389/fphys.2013.00026 PMC3576706

[28] Fiskum C, Andersen TG, Bornas X, Aslaksen PM, Flaten MA, Jacobsen K. Non-linear heart rate variability as a discriminator of internalizing psychopathology and negative affect in children with internalizing problems and healthy controls. Front Physiol. 2018;9:561. 10.3389/fphys.2018.00561. 29875679 10.3389/fphys.2018.00561 PMC5974559

[29] Perkiömäki JS. Heart rate variability and non-linear dynamics in risk stratification. Front Physiol. 2011;2:81. 10.3389/fphys.2011.00081. 22084633 10.3389/fphys.2011.00081 PMC3210967

[30] Chattopadhyay S. The importance of time-domain HRV analysis in cardiac health prediction. Series Cardiol Res 2022; 4: 19–23. 10.54178/2768-5985.2022a5.

[31] Li K, Rüdiger H, Ziemssen T. Spectral analysis of heart rate variability: time window matters. Front Neurol. 2019;10:545. 10.3389/fneur.2019.00545. 31191437 10.3389/fneur.2019.00545 PMC6548839

[32] Lim CX, O’Brien WH, Watford TS, Suvanbenjakule P. Psychological inflexibility and HF-HRV reactivity to laboratory stressors. J Contextual Behav Sci. 2022;26:134–8. 10.1016/j.jcbs.2022.09.004.

[33] Tharion E, Parthasarathy S, Neelakantan N. Short-term heart rate variability measures in students during examinations. Natl Med J India. 2009;22(2):63–6. 19852338

[34] Visnovcova Z, Mestanik M, Javorka M, Mokra D, Gala M, Jurko A, et al. Complexity and time asymmetry of heart rate variability are altered in acute mental stress. Physiol Meas. 2014;35(7):1319–34. 10.1088/0967-3334/35/7/1319. 24854052 10.1088/0967-3334/35/7/1319

[35] Taelman J, Vandeput S, Vlemincx E, Spaepen A, Van Huffel S. Instantaneous changes in heart rate regulation due to mental load in simulated office work. Eur J Appl Physiol. 2011;111(7):1497–505. 10.1007/s00421-010-1776-0. 21188414 10.1007/s00421-010-1776-0

[36] Adjei T, von Rosenberg W, Nakamura T, Chanwimalueang T, Mandic DP. The ClassA framework: HRV based assessment of SNS and PNS dynamics without LF-HF controversies. Front Physiol. 2019;10:505. 10.3389/fphys.2019.00505. 31133868 10.3389/fphys.2019.00505 PMC6511892

[37] Schubert C, Lambertz M, Nelesen RA, Bardwell W, Choi JB, Dimsdale JE. Effects of stress on heart rate complexity—a comparison between short-term and chronic stress. Biol Psychol. 2009;80(3):325–32. 10.1016/j.biopsycho.2008.11.005. 19100813 10.1016/j.biopsycho.2008.11.005 PMC2653595

[38] Dedovic K, Renwick R, Mahani NK, Engert V, Lupien SJ, Pruessner JC. The Montreal imaging stress task: using functional imaging to investigate the effects of perceiving and processing psychosocial stress in the human brain. J Psychiatry Neurosci. 2005;30(5):319–25. 16151536 PMC1197276

[39] Zysset S, Müller K, Lohmann G, von Cramon DY. Color-word matching stroop task: separating interference and response conflict. Neuroimage. 2001;13(1):29–36. 10.1006/nimg.2000.0665. 11133306 10.1006/nimg.2000.0665

[40] Ursin H, Eriksen HR. The cognitive activation theory of stress. Psychoneuroendocrinology. 2004;29(5):567–82. 10.1016/S0306-4530(03)00091-X. 15041082 10.1016/S0306-4530(03)00091-X

[41] Alessa FM, Alhaag MH, Al-harkan IM, Nasr MM, Kaid H, Hammami N. Evaluating physical stress across task difficulty levels in augmented reality-assisted industrial maintenance. Appl Sci. 2024;14(1):363. 10.3390/app14010363.

[42] Bernardi L, Porta C, Gabutti A, Spicuzza L, Sleight P. Modulatory effects of respiration. Auton Neurosci. 2001;90(1–2):47–56. 10.1016/S1566-0702(01)00267-3. 11485292 10.1016/S1566-0702(01)00267-3

[43] Hart SG, Staveland LE. Development of NASA-TLX (Task Load Index): results of empirical and theoretical research. In: Hancock PA, Meshkati N, eds. Advances in psychology: Human mental workload. Vol. 52. North-Holland, 1988: 139–183. 10.1016/S0166-4115(08)62386-9.

[44] Niskanen JP, Tarvainen MP, Ranta-Aho PO, Karjalainen PA. Software for advanced HRV analysis. Comput Methods Programs Biomed. 2004;76(1):73–81. 10.1016/j.cmpb.2004.03.004. 15313543 10.1016/j.cmpb.2004.03.004

[45] Feldman AM. Modulation of adrenergic receptors and G-transduction proteins in failing human ventricular myocardium. Circulation 1993; 87(5 Suppl): IV27–IV34. 8485831

[46] McCraty R, Shaffer F. Heart rate variability: new perspectives on physiological mechanisms, assessment of self-regulatory capacity, and health risk. Glob Adv Health Med. 2015;4(1):46–61. 10.7453/gahmj.2014.073. 25694852 10.7453/gahmj.2014.073 PMC4311559

[47] van Paridon KN, Timmis MA, Nevison CM, Bristow M. The anticipatory stress response to sport competition: a systematic review with meta-analysis of cortisol reactivity. BMJ Open Sport Exerc Med. 2017;3(1):e000261. 10.1136/bmjsem-2017-000261. 29177073 10.1136/bmjsem-2017-000261 PMC5604718

[48] De Rivecourt M, Kuperus MN, Post WJ, Mulder LJM. Cardiovascular and eye activity measures as indices for momentary changes in mental effort during simulated flight. Ergonomics. 2008;51(9):1295–319. 10.1080/00140130802120267. 18802817 10.1080/00140130802120267

[49] Storniolo JL, Cairo B, Porta A, Cavallari P. Symbolic analysis of the heart rate variability during the plateau phase following maximal sprint exercise. Front Physiol. 2021;12:632883. 33833687 10.3389/fphys.2021.632883 PMC8021730

[50] Dallman MF, Jones MT. Corticosteroid feedback control of ACTH secretion: effect of stress-induced corticosterone secretion on subsequent stress responses in the rat. Endocrinology. 1973;92(5):1367–75. 10.1210/endo-92-5-1367. 4348647 10.1210/endo-92-5-1367

[51] Belda X, Fuentes S, Daviu N, Nadal R, Armario A. Stress-induced sensitization: the hypothalamic-pituitary-adrenal axis and beyond. Stress. 2015;18(3):269–79. 10.3109/10253890.2015.1067678. 26300109 10.3109/10253890.2015.1067678

[52] Kim HG, Cheon EJ, Bai DS, Lee YH, Koo BH. Stress and heart rate variability: a meta-analysis and review of the literature. Psychiatry Investig. 2018;15(3):235–45. 10.30773/pi.2017.08.17. 29486547 10.30773/pi.2017.08.17 PMC5900369

[53] Thayer JF, Ahs F, Fredrikson M, Sollers JJ, Wager TD. A meta-analysis of heart rate variability and neuroimaging studies: implications for heart rate variability as a marker of stress and health. Neurosci Biobehav Rev. 2012;36(2):747–56. 10.1016/j.neubiorev.2011.11.009. 22178086 10.1016/j.neubiorev.2011.11.009

[54] Ali MK, Liu L, Hussain A, Zheng D, Alam M, Chen JH, et al. Root mean square of successive differences is not a valid measure of parasympathetic reactivity during slow deep breathing. Am J Physiol Regul Integr Comp Physiol. 2023;324(4):R446–56. 10.1152/ajpregu.00272.2022. 36717167 10.1152/ajpregu.00272.2022

[55] Minarini G. Root mean square of the successive differences as marker of the parasympathetic system and difference in the outcome after ANS stimulation. In: Aslanidis T, ed. Autonomic nervous system monitoring – Heart rate variability. IntechOpen, 2020. 10.5772/intechopen.89827.

[56] Fisher AJ, Song J, Soyster PD. Toward a systems-based approach to understanding the role of the sympathetic nervous system in depression. World Psychiatry. 2021;20(2):295–6. 10.1002/wps.20872. 34002517 10.1002/wps.20872 PMC8129862

[57] Weissman DG, Mendes WB. Correlation of sympathetic and parasympathetic nervous system activity during rest and acute stress tasks. Int J Psychophysiol. 2021;162:60–8. 10.1016/j.ijpsycho.2021.01.015. 33561515 10.1016/j.ijpsycho.2021.01.015 PMC7987796

